# Tumor metabolic and secondary lymphoid organ metabolic markers on 18F-fludeoxyglucose positron emission tomography predict prognosis of immune checkpoint inhibitors in advanced lung cancer

**DOI:** 10.3389/fimmu.2022.1004351

**Published:** 2022-10-21

**Authors:** Peng Jin, Menglin Bai, Jie Liu, Jinming Yu, Xue Meng

**Affiliations:** ^1^ Department of Radiation Oncology and Shandong Provincial Key Laboratory of Radiation Oncology, Shandong Cancer Hospital and Institute, Shandong First Medical University and Shandong Academy of Medical Sciences, Jinan, China; ^2^ Department of Radiation Oncology, Shandong University Cancer Center, Jinan, China; ^3^ Research Unit of Radiation Oncology, Chinese Academy of Medical Sciences, Jinan, China

**Keywords:** FDG-PET/CT, prognosis, immune checkpoint inhibitors, metabolic tumor burden, secondary lymphoid organs

## Abstract

**Background:**

The purpose of this study was to investigate the predictive value of tumor metabolic parameters in combination with secondary lymphoid metabolic parameters on positron emission tomography (PET)/computed tomography (CT) for immune checkpoint inhibitor (ICI) prognosis in advanced lung cancer.

**Methods:**

This study retrospectively included 125 patients who underwent 18F-fludeoxyglucose (FDG) PET/CT before ICI therapy, including 41 patients who underwent a second PET/CT scan during ICI treatment. The measured PET/CT parameters included tumor metabolism parameters [maximum standardized uptake value (SUVmax), mean standardized uptake value (SUVmean), total lesion glycolysis (TLG), and total metabolic tumor volume (TMTV)] and secondary lymphoid organ metabolism parameters [spleen-to-liver SUVmax ratio (SLR) and bone marrow-to-liver SUVmax ratio (BLR)]. The correlation of PET/CT metabolic parameters with early ICI treatment response, progression-free survival (PFS), and overall survival (OS) was analyzed.

**Results:**

Within a median follow-up of 28.7 months, there were 44 responders and 81 non-responders. The median PFS was 8.6 months (95% confidence interval (CI): 5.872–11.328), and the median OS was 20.4 months (95% CI: 15.526–25.274). Pretreatment tumor metabolic parameters were not associated with early treatment responses. The high bone marrow metabolism (BLR >1.03) was significantly associated with a shorter PFS (p = 0.008). Patients with a high TMTV (>168 mL) and high spleen metabolism (SLR >1.08) had poor OS (p = 0.019 and p = 0.018, respectively). Among the 41 patients who underwent a second PET/CT scan, the ΔSUVmax was significantly lower (p = 0.01) and the SLR was significantly higher (p = 0.0086) in the responders. Populations with low-risk characteristics (low TMTV, low SLR, and ΔSLR > 0) had the longest survival times.

**Conclusion:**

High pretreatment TMTV and SLR are associated with poor OS, and increased spleen metabolism after ICI therapy predicts treatment benefit. This indicates that the combination of tumor and spleen metabolic parameters is a valuable prognostic strategy.

## Introduction

Immune checkpoint inhibitors (ICIs) have been widely used in cancer treatment in recent years. In advanced lung cancer, ICIs and their combination regimens as standard first- and second-line treatments have significantly improved survival (1–3). However, although the use of ICIs has met with great success, only a small proportion of patients achieve long-term survival benefits (4, 5). Therefore, many studies have explored predictors to assess ICI response and prognosis to improve early assessment of patient conditions and guide further treatment. Molecular imaging, as a non-invasive imaging method, is highly useful in monitoring tumor response. The most widely used molecular imaging technology in clinical practice is 2-[^18^F]fluoro-2-deoxy-D-glucose (2-[^18^F]FDG) positron emission tomography/computed tomography (PET/CT) (6). Pretreatment 2-[^18^F]FDG PET/CT has been proven to predict the treatment effect and prognosis of patients with cancer through measurement of various metabolic parameters (7). Among them, volume and metabolic uptake parameters, as two important predictive markers, have shown different predictive values in many studies. In patients with advanced non-small cell lung cancer (NSCLC) receiving first-line programmed cell death protein 1 (PD1) therapy, pretreatment total metabolic tumor volume (TMTV) and mean standardized uptake value (SUVmean) are predictive of better tumor responses, and high TMTV is significantly associated with poor overall survival (OS) (8).

It may be difficult to accurately distinguish patients with different prognoses using a single metabolic parameter index; tumor characteristics alone cannot comprehensively predict prognoses. Combined biological parameters have a better predictive capability. In patients with advanced NSCLC, the combination of tumor burden (TMTV) and inflammatory status parameters (derived neutrophil-to-lymphocytes ratio) can predict poor survival after ICI therapy (9). Importantly, the selection of suitable candidates requires an understanding of the physiological response mechanism of immunotherapy and compatibility with the biological properties of immunotherapy. A systemic antitumor response involving complex immune cell interactions in the primary and secondary lymphoid organs is required for successful immunotherapy (10). The immune cell composition of the host prior to immunotherapy influences the therapeutic response (11). Therefore, a combination of tumor and lymphoid PET/CT metabolic parameters may better reflect the immune status of the human body and response to immunotherapy. Studies have shown that in patients with melanoma, higher TMTV and bone marrow and spleen metabolism than normal before ICI treatment are correlated with a low survival rate. Using pretreatment tumor metabolic parameter data in combination with bone marrow metabolic parameters can achieve more detailed patient stratification (12).

PET/CT can be used to monitor systemic immune activation following immunotherapy (13). Immunotherapy induces immune cell activation, including changes in the lymphocyte proliferative status, influx and recruitment of dendritic cells (DCs) and macrophages, metabolic reprogramming of activated peripheral T cells, and increased glycolysis following myeloid cell activation. These changes lead to enhanced glucose metabolism in lymphoid organs, and FDG PET/CT can capture complex immune cell changes and changes in metabolic patterns in lymphoid organs (13). Therefore, the combination of pretreatment tumor and lymphoid organ metabolic biomarkers in the host immune environment and the status of immune cells in the lymphoid organs in response to immunotherapy can better predict patient response and prognosis. This study aimed to evaluate the correlation of tumor and secondary lymphoid organ (spleen and bone marrow) metabolism with patient survival, as well as the impact of posttreatment changes in these metabolic parameters on patient prognosis.

## Materials and methods

### Study design and participants

This retrospective study evaluated patients with advanced lung cancer who received ICI therapy at Shandong Cancer Hospital between February 2017 and November 2020 and underwent 18F-FDG PET/CT before ICI treatment. The inclusion criteria were as follows (1): biopsy-proven NSCLC or SCLC (2); stage IV disease according to the American Joint Committee on Cancer 8th edition staging (3); received ICI treatment for more than 4 cycles; and (4) age >18 years. The exclusion criteria were as follows (1): more than 8 weeks’ interval between pretreatment PET/CT and first ICI treatment (2); treatment with corticosteroids or granulocyte-macrophage colony-stimulating factor over the past 2 months (3); splenic metastasis (4); acute or chronic infections or autoimmune disorders; and (5) other primary malignancies. A total of 125 patients were included in this study. Among them, 41 patients underwent a second PET/CT examination after the ICI treatment.

This study was approved by the institutional review board of Shandong Cancer Hospital and complied with the Declaration of Helsinki.

### PET/CT protocol

All 18F-FDG PET/CT scans were performed at the Department of Nuclear Medicine of Shandong Cancer Hospital. FDG [370 MBq (10 mCi)] was administered intravenously after a 6 h-fast and at a blood glucose level of <11 mol/L. The time interval between the injection and image scanning was 60 min. All patients underwent a 5 min-whole-body emission scan from the base of the skull to the mid-femur. During the PET/CT scan, all patients were asked to remain calm and breathe slowly; 18F-FDG PET/CT was performed using a PET/CT scanner (GEMINI TF Big Bore; Philips Healthcare) and at a thickness of 4.25 mm/slice in the axial direction. Images from multi-slice CT scans were attenuation corrected and reconstructed. PET, CT, and fused PET/CT images were presented in coronal, sagittal, and transverse slices and viewed on a Xeleris workstation (GE Healthcare).

### Analysis of PET/CT data

PET/CT images were analyzed using MIM software (version 7.1.7 Cleveland, OH, USA). Two experienced nuclear medicine physicians performed 18F-FDG PET/CT according to the standardized protocol. In PET/CT images, an SUV of 2.5 was set as the threshold, and a region of interest was automatically drawn over hypermetabolic lesions. All metastatic lesions with a high FDG uptake were analyzed. Patients with physiological hypermetabolic lesions or lesions that were considered inflammatory were excluded. The SUVmax, SUVmean, total lesion glycolysis (TLG) values, and metabolic tumor volume (MTV) of each lesion were measured using MIM software, and the MTV of all lesions was recorded as the TMTV. The spleen and bone marrow were delineated as secondary lymphoid organs for the analysis. After excluding patients with splenic metastases, the entire spleen was completely delineated to analyze the spleen metabolism indices.

Physiological hypermetabolism in the kidney adjacent to the spleen was avoided during delineation. To better reflect the function of bone marrow metabolism, we improved the identification of bone marrow. The delineation included the L1 to L4 (lumbar) vertebral bodies, entire pelvis, and upper end of the femur. The bone cortex signal was then identified according to the region grow function, and all cortical bones in this region were automatically delineated. The area after deducting the bone cortex was regarded as the bone marrow, and information on the bone marrow area was obtained (**Supplementary Figure 1**). The functions of the bone marrow and spleen were accurately reflected by normalization of their parameter values to those of the liver. Liver SUVmax values were averaged by obtaining the SUVmax value of three 1 cm-spherical volumes of interest in the liver. The bone marrow and spleen SUVmax values were divided by the liver SUVmax value to obtain the bone marrow-to-liver ratio (BLR) and spleen-to-liver ratio (SLR). All patients with bone metastases were excluded from the bone marrow signal analysis. The interval changes in the PET/CT parameters were defined as ΔSUVmax, ΔSUVmean, ΔTLG, ΔTMTV, ΔSLR, and ΔBLR.

### Assessment of patient response to ICI treatment and prognosis

Patients were followed up clinically and radiologically every 6 weeks. Patient response to treatment was assessed according to the Response Evaluation Criteria in Solid Tumors (version 1.1) (14). Treatment response was classified as complete response, partial response, stable disease, or progressive disease. To assess early response to ICI treatment, patients who achieved partial response or complete response at any time during the first four cycles of ICI treatment were considered as responders. In contrast, patients who had stable disease or progressive disease within the first four cycles of ICI treatment were considered as non-responders. Patient outcomes were assessed using overall survival (OS) and progression-free survival (PFS). OS was defined as the length of time from the date of the first ICI treatment to the date of any-cause death or the date when the patient was alive at the last available follow-up. PFS was defined as the length of time from the date of the first ICI treatment to the date of tumor progression or any-cause death.

### Statistical analysis

Differences in PET biomarker values among different groups were compared using the Mann–Whitney U test. Continuous variables used for comparison are described as mean ± standard deviation. With the median values of metabolic parameters as cutoff points, the survival prognostic value of all PET/CT parameter biomarkers was investigated using the Cox survival model. Multivariate analyses of significant prognostic factors in univariate analysis were performed in a stepwise manner using Cox proportional hazards regression models to identify independent risk factors. Survival curves were generated using the Kaplan–Meier method and compared between groups using the log-rank test. All statistical analyses were performed using the IBM SPSS statistical software (version 26) and GraphPad Prism (version 9). Statistical significance was set at p < 0.05.

## Results

### Patient characteristics

The median patient age was 66 years (range, 30–81 years), and there were 106 patients with NSCLC (37 and 69 patients with squamous and non-squamous cell carcinoma, respectively) and 19 patients with SCLC. There were no significant differences in PET/CT metabolic parameter values between patients with NSCLC and SCLC (**Supplementary Table 1**). **Table 1** lists the patient characteristics. All patients received ICI therapy; of them, 41 patients received first-line ICI alone or ICI combined with chemotherapy, and 84 patients received ≥2 lines of ICI alone or ICI combined with chemotherapy. The median follow-up period was 28.7 months (range, 1.367–39.933 months). During the follow-up period, 106 patients progressed and 76 patients died. In total, 48 patients were tested for programmed death-ligand 1 (PD-L1) expression by immunohistochemistry, of which 39 and 9 patients were positive and negative, respectively. A second PET/CT scan was performed during ICI treatment in 41/125 patients. The median time between the second PET/CT scan and start of ICI treatment was 4.25 months (range, 1.4–8.1 months).

**Table 1:** Patient characteristics.

Characteristic	Median [range], n (%)
Age	66 [30-81]
Sex	
Male	85 (68)
Female	40 (32)
Smoking history	
Smoking	51 (40.8)
Non-smoking	74 (59.2)
BMI	
<25	76 (60.8)
≥25	49 (39.2)
Histological variant	
Small cell lung cancer	19 (15.2)
Squamous cell carcinoma	37 (29.6)
Non-squamous cell carcinoma	69 (55.2)
Immunotherapy	
First line	41 (32.8)
≥Second line	
Platinum based CT + Pemetrexed (or Paclitaxel)	30 (24)
Platinum based CT + Pemetrexed (or Paclitaxel) + Bevacizumab	35 (28)
Platinum based CT + Etoposide	19 (15.2)
Drugs	
Nivolumab	45 (36)
Pembrolizumab	61 (48.8)
Atezolizumab	8 (6.4)
Durvalumab	11 (8.8)
PD-L1 expression	
Positive	39 (31.2)
Negative	9 (7.2)
Unknown	77 (61.6)

BMI, Body Mass Index; CT, chemotherapy; PD-L1, programmed death ligand 1.

### Relationship between pretreatment PET biomarkers and immune responses

Among the 125 patients, 44 patients were responders (33.6%, 43 and 1 patient with partial response and complete response, respectively) and 81 patients were non-responders (66.4%, 49 and 32 patients with stable disease and progressive disease, respectively). There were no significant differences in pretreatment SUVmax, SUVmean, TLG, and TMTV values between responders and non-responders (**Table 2**). With respect to the metabolism indices of secondary lymphoid organs, there were no significant between-group differences in SLR and BLR (**Table 2**).

**Table 2:** Comparison of pretreatment PET/CT metabolic parameters between response groups.

	Responder (n = 44)	Non-responders (n = 81)	P
Tumor metabolism			
SUVmax	12.31 ± 5.58	10.83 ± 6.08	0.099
SUVmean	4.82 ± 1.39	4.45 ± 1.68	0.067
TLG	325.92 ± 401.56	480.45 ± 764.95	0.992
TMTV	159.60 ± 169.08	173.65 ± 250.09	0.587
Secondary lymphoid organ metabolism			
SLR	1.08 ± 0.23	1.09 ± 0.20	0.408
BLR	1.00 ± 0.23	1.07 ± 0.28	0.122

SUVmax, maximum standardized uptake value; SUVmean, mean standardized uptake value; TLG, total lesion glycolysis; TMTV, total metabolic tumor volume; SLR, spleen-to-liver SUVmax ratio; BLR, bone marrow-to-liver SUVmax ratio.

### PET metabolic parameters were correlated with survival

Regarding the relationship between PET/CT metabolic parameters and the prognosis of patients with advanced lung cancer treated with ICI, the median PFS was 8.6 months (95% confidence interval (CI): 5.872–11.328). The results of the univariate analysis showed that there was no significant correlation among pretreatment SUVmax, SUVmean, TLG, and TMTV and PFS. Analysis of secondary lymphoid organs revealed that high bone marrow metabolism (BLR >1.03) was significantly associated with a shorter PFS (p = 0.008) (**Supplementary Table 2**). Survival analysis showed that patients with BLR >1.03 had a shorter PFS (**Supplementary Figure 2**).

**Figure 1:**
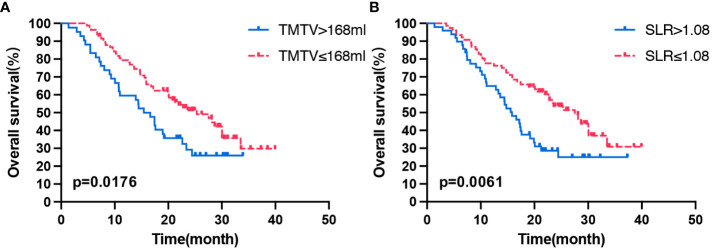
Kaplan–Meier curves of OS according to the TMTV **(A)** and SLR **(B)**. OS, overall survival; TMTV, total metabolic tumor volume; SLR, spleen-to-liver SUVmax ratio.

At the time of analysis, 76 patients (60.8%) had died in the overall population, and the median OS was 20.4 months (95% CI: 15.526–25.274). Patients with a high TMTV (>168 mL) and high spleen metabolism (SLR >1.08) had poor OS (p = 0.019, p = 0.018). In the multivariate analysis, high TMTV and spleen metabolism were independent prognostic factors for OS (hazard ratio (HR): 1.604, p = 0.048 and HR: 1.603, p = 0.045, respectively) (**Table 3**). Analysis by Kaplan–Meier curves confirmed that patients with TMTV > 168 mL and SLR >1.08 had shorter OS (**Figure 1**). Thus, high TMTV correlates significantly with OS in multivariate analysis; for secondary lymphoid organs, although high bone marrow metabolism correlates with disease progression, it does not correlate with OS in the same manner as high spleen metabolism.

**Table 3:** Prognostic significance of PET biomarkers for overall survival in univariate and multivariate analyses.

	Overall survival			
	Univariate	P	Multivariate	P
	HR (95% CI)		HR (95% CI)	
Tumor metabolism				
High SUVmax	0.990 (0.628-1.561)	0.965	–	–
High SUVmean	0.784 (0.498-1.236)	0.295	–	–
High TLG	1.580 (0.986-2.531)	0.057	–	–
High TMTV	1.737 (1.095-2.756)	0.019	1.604 (1.003-2.563)	0.048
Secondary lymphoid organ metabolism				
High SLR	1.728 (1.097-2.721)	0.018	1.603 (1.011-2.541)	0.045
High BLR	1.418 (0.857-2.346)	0.174	–	–

HR, hazard ratio; SUVmax, maximum standardized uptake value; SUVmean, mean standardized uptake value; TLG, total lesion glycolysis; TMTV, total metabolic tumor volume; SLR, spleen-to-liver SUVmax ratio; BLR, bone marrow-to-liver SUVmax ratio.

### Changes in PET metabolic parameters were correlated with immune responses

**Figure 2:**
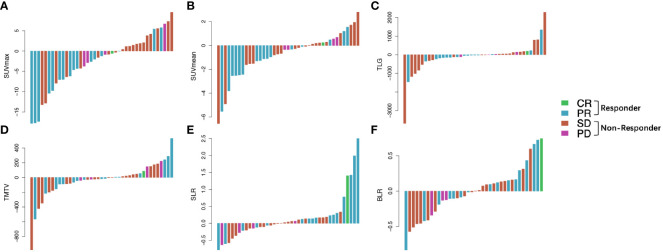
Waterfall plots of the ΔSUVmax **(A)**, ΔSUVmean **(B)**, ΔTLG **(C)**, ΔTMTV **(D)**, ΔSLR **(E)**, and ΔBLR **(F)** before and after ICI treatment according to treatment response. Responder: PR and CR; non-responder, SD and PD. Δ indicates interval changes in the PET/CT parameters of SUVmax: maximum standardized uptake value; SUVmean, mean standardized uptake value; TLG, total lesion glycolysis; TMTV, total metabolic tumor volume; SLR, spleen-to-liver SUVmax ratio; BLR, bone marrow-to-liver SUVmax ratio; ICI, immune checkpoint inhibitors; PR, partial response; CR, complete response; SD, stable disease; PD, progressive disease.

In total, 41 patients underwent a second 18F-FDG PET/CT scan after ICI treatment, and the median interval between the start of ICI treatment and second PET/CT scan was 4.25 months (range: 1.4–8.1 months). For analyzing the relationship between changes in PET/CT metabolic parameters and the early response to ICI therapy, waterfall plots of ΔSUVmean, ΔSUVmax, ΔTLG, ΔTMTV, ΔSLR, and ΔBLR in patients with different treatment responses were constructed (**Figure 2**). Decreases in SUVmean, SUVmax, TLG, and TMTV values were observed in both responders and non-responders. Compared with the non-responder group, the responder group showed a significantly lower ΔSUVmax (p = 0.01) (**Supplementary Table 3**). Metabolic changes in the spleen and bone marrow were also different between responders and non-responders. SLR and BLR were both increased after ICI treatment in responders. ΔSLR values were significantly higher in responders than in non-responders (0.45 ± 0.89 and −0.75 ± 0.23, respectively, p = 0.02). Meanwhile, there was no significant between-group difference in ΔBLR (**Supplementary Table 3**).

### Changes in PET metabolic parameters was correlated with survival

**Figure 3:**
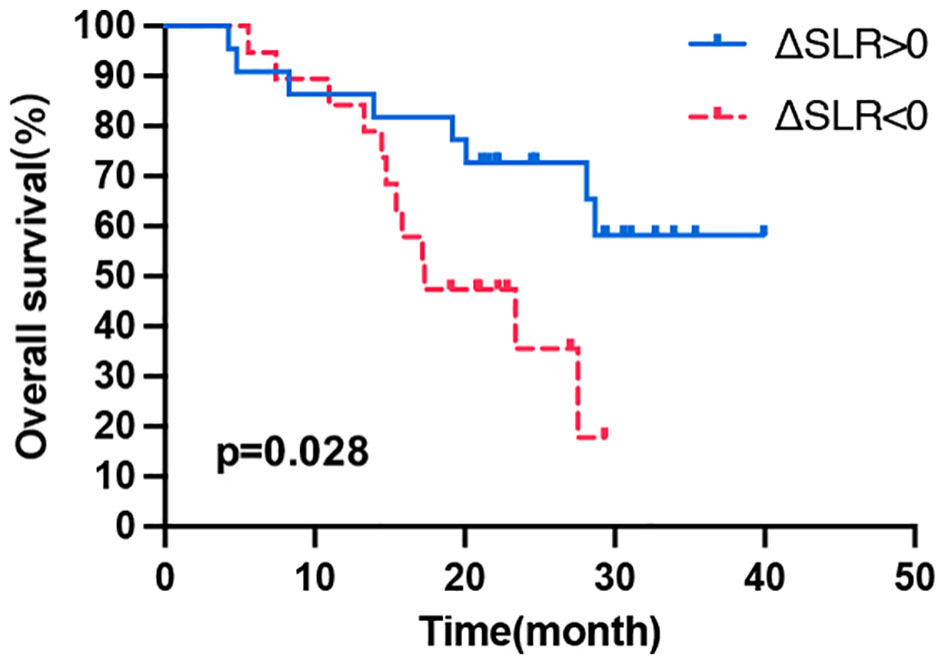
Kaplan–Meier curves of OS according to the ΔSLR. OS, overall survival; Δ indicates interval changes in the PET/CT parameter of SLR, spleen-to-liver SUVmax ratio.

With respect to the correlation between changes in PET/CT metabolic parameters after ICI treatment and patient prognosis, all values (ΔSUVmean, ΔSUVmax, ΔTLG, ΔTMTV, ΔSLR, or ΔBLR) were not correlated with PFS (**Table 4**). Then, the patients were divided into long- and short-term survival populations based on the median OS of the total population. The results showed that the ΔSUVmean, ΔSUVmax, ΔTLG, and ΔTMTV values were not significantly different between the long- and short-term survival populations. Regarding the secondary lymphoid organs, ΔSLR in the long-term survival population was significantly higher than that in the short-term survival population (p = 0.005), while ΔBLR was not significantly different between the two populations (**Supplementary Table 3**). Univariate analysis of influencing factors of OS revealed that a ΔSLR > 0 was significantly associated with a longer OS (p = 0.034; **Table 4**). Kaplan–Meier curves showed that patients with ΔSLR > 0 had a longer OS (**Figure 3**). Thus, increased spleen metabolism significantly correlated with a longer OS.

**Table 4:** Univariate analyses of prognostic significance of ΔSUVmean, ΔSUVmax, ΔTLG, ΔTMTV, ΔSLR, and ΔBLR in progression-free survival and overall survival.

	Progression-free survival	P	Overall survival	P
	HR (95% CI)		HR (95% CI)	
Tumor metabolism				
ΔSUVmax>-2.57	1.085 (0.520-2.263)	0.828	1.366 (0.558-3.345)	0.495
ΔSUVmean>-0.78	1.175 (0.564-2.449)	0.667	1.991 (0.762-5.203)	0.160
ΔTLG>-111	0.629 (0.295-1.344)	0.232	0.726 (0.297-1.772)	0.482
ΔTMTV>-36	0.784 (0.365-1.686)	0.534	0.623 (0.257-1.506)	0.293
Secondary lymphoid organ metabolism				
ΔSLR>0	0.488 (0.236-1.009)	0.053	0.361 (0.141-0.926)	0.034
ΔBLR>0	0.550 (0.256-1.182)	0.126	0.496 (0.183-1.342)	0.167

HR, hazard ratio; SUVmax: maximum standardized uptake value; SUVmean, mean standardized uptake value; TLG, total lesion glycolysis; TMTV, total metabolic tumor volume; SLR, spleen-to-liver SUVmax ratio; BLR, bone marrow-to-liver SUVmax ratio.

### Tumor volume combined with secondary lymphoid organ metabolism can better predict prognosis

**Figure 4:**
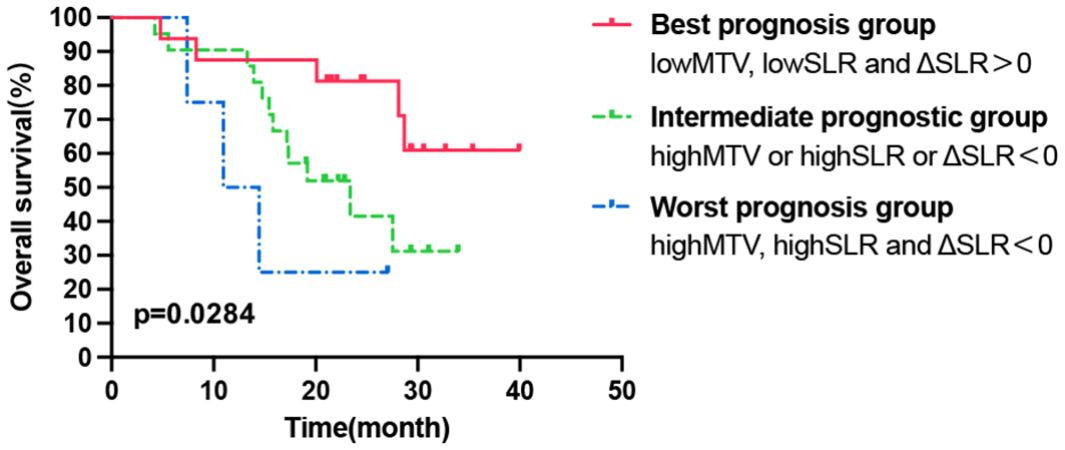
Kaplan–Meier curve of OS according to the combination of tumor and secondary lymphoid organ metabolic markers. OS, overall survival.

According to the above results, high pretreatment TMTV and SLR values were significantly correlated with shorter OS, while ΔSLR > 0 post-ICI treatment predicted a better OS. Combining these metabolic biomarkers may better reflect the tumor burden, basal immune status, and immune activation response to ICI treatment, and thus, the prognosis can be more accurately classified. The patients who underwent a second PET/CT scan after treatment were divided into three groups (1): the worst prognosis group with high tumor burden, poor immune status, and ineffective immune activation (high TMTV, high SLR, and ΔSLR < 0) (2); the best prognosis group with a low tumor burden, better basic immune status, and effective immune activation (low TMTV, low SLR, and ΔSLR > 0); and (3) the intermediate prognostic group (high TMTV or high SLR or ΔSLR < 0). As shown in **Figure 4**, there were significant differences in OS among the three populations, and the best prognosis group had the most significant survival benefit.

## Discussion

The current study shows that the 18F-FDG PET/CT metabolic parameter TMTV and metabolic parameters of secondary lymphoid organs, especially those of the spleen, have a significant value in predicting the early treatment response and prognosis of ICI in advanced lung cancer. This result is theoretically based on the biological properties of immunotherapy. In addition to the tumor burden, the immune system status and physiological response mechanisms to immunotherapy were also considered.

Baseline FDG PET/CT metabolic indicators can be used to predict the efficacy of lung cancer immunotherapy. In this study, TMTV, which reflects tumor burden, predicted patient prognosis; high pretreatment TMTV was significantly associated with a poor median OS, consistent with the results of a previous study (15). A high MTV is a significant prognostic factor in patients with lung cancer (16). Some studies have also shown that SUVmax predicts prognosis in patients with lung cancer (17). In the era of immunotherapy for lung cancer, tumor volume metabolic parameters appear to better reflect prognosis than semi-quantitative parameters, such as SUVmax. Retrospective studies showed that TLG and MTV, but not SUVmax, are independent predictors of disease progression and survival after anti-PD-1 therapy in patients with lung cancer (18, 19). A recent prospective study also affirmed the reliable status of high MTV in predicting prognosis of ICIs in NSCLC (20).

In recent years, many studies have explored the combined use of multiple metabolic markers, such as hematological parameters. Analysis of TMTV combined with the derived neutrophil-to-lymphocyte ratio can predict treatment responses and OS after ICI treatment in patients with advanced NSCLC (9). Matteo et al. also revealed the predictive effect of MTV combined with systemic inflammation degree on NSCLC ICI therapy (21). The human immune system is involved in many complex responses. In addition to tumors, complex markers in the circulation or lymphoid organs can influence the outcomes of immunotherapy. Our study combined tumor and secondary lymphoid organ metabolic parameters, such as pretreatment TMTV, SLR, and ΔSLR as indicators of immune activation status, and established a new method to predict the prognosis of advanced lung cancer with ICI therapy. Thus, the patients can be stratified more accurately.

High FDG uptake in lymphoid organs is closely associated with host inflammatory activity and the degree of the systemic immune response associated with tumor activity (22, 23). Studies have also demonstrated significant associations between FDG PET/CT metabolic parameters and populations of various immune cells in the tumor microenvironment (e.g., regulatory T cell (Tregs), tumor-associated macrophages, DCs, and tumor-infiltrating lymphocytes (24). The high baseline uptake of FDG appears to be largely due to the immunosuppressive cells within them. As Shimura et al. recently discovered a mechanism by which myeloid-derived suppressor cells (MDSCs) in the bone marrow might trigger increased bone marrow FDG uptake, leading to poor patient outcomes (25). Increased FDG uptake by 18F-FDG PET/CT indicates a premetastatic niche mediated by MDSCs in the lymph nodes (26). Our results showed that pretreatment high bone marrow metabolism (BLR >1.03) was significantly associated with a shorter PFS, whereas pretreatment high spleen metabolism (SLR >1.08) was an independent risk factor for poor OS.

The spleen is the largest secondary lymphoid organ, and splenic adherent cells release factors to promote immunosuppressive cell maturation and subsequent migration into the circulation (27). The spleen and bone marrow accumulate a large number of Tregs and MDSCs that migrate to the peripheral lymphoid organs and tumor sites, contributing to the formation of an immunosuppressive tumor microenvironment (28–30). Immature monocytes and granulocytes produced by the bone marrow migrate into the tumor microenvironment and then differentiate into tumor-associated macrophages and tumor-associated neutrophils, respectively, exerting suppressive immune functions (31). Therefore, pretreatment high SLR and BLR may be representative of immune organs that are enriched with mobilized tumor-associated immunosuppressive cells, mediating a suppressive immune microenvironment. This systemic baseline immunosuppressive state leads to a state of relative resistance to ICI therapy.

This study found that ΔSLR values were significantly higher in responders and elevated ΔSLR was significantly correlated with a longer survival. The guidelines on 18F-FDG PET/CT jointly proposed by European Association of Nuclear Medicine (EANM)/Society of Nuclear Medicine and Molecular Imaging (SNMMI)/Australian and New Zealand Society of Nuclear Medicine (ANZSNM) pointed out that the increase splenic uptake after immunotherapy is mostly due to the release of T lymphocytes, resulting in a better prognosis (32). This is consistent with the results of this study. Immunotherapy triggers complex systemic immune activation involving changes in multiple immune cell populations in secondary lymphoid organs, increased immune cell proliferation and influx, recruitment of DCs and macrophages, and activation of T cells, all of which are accompanied by cellular metabolic patterns, leading to increased glycolytic metabolism in lymphoid organs. FDG PET/CT imaging enables the visualization of changes in the functions of immune cells and their status in the bone marrow and spleen to monitor systemic immune responses (13). Elevated FDG uptake in lymphoid organs indicates a potent response to ICI treatment and is more likely to occur in patients with a low immunosuppressive status. After ICI immune activation, the metabolic pattern of peripheral lymphoid organs is mainly influenced by effector immune cells rather than suppressor immune cells. The metabolic reprogramming of T lymphocytes (33) and activation of DCs increase glycolysis (34). Additionally, ICI treatment reverses inhibition of CD28-mediated metabolic reprogramming of lymphocyte activation by PD-1 and improves T-cell glycolysis (35). As such, the high spleen metabolism after ICI treatment indicates that effective immune activation of various lymphocytes results in a good immune response and long-term survival benefit.

The use of 18F-FDG PET/CT to effectively predict ICI responses has been validated in several studies. Increased splenic FDG uptake was observed in responders treated with anti-PD1 in patients with Hodgkin lymphoma (36). In patients with melanoma treated with ipilimumab, sarcoid-like lymphadenopathy was detected with an early response on 18F-FDG PET/CT (37). A mouse model showed increased FDG uptake in the spleen after 4 weeks of anti-PD-L1/LAG-3 immunotherapy, and immune cells could rapidly switch to extensive glycolysis after activation, accompanied by elevated neutrophil infiltration (38). A retrospective analysis also reported increased FDG uptake in the spleen and bone marrow of patients with an effective immune response to CTLA-4 or PD-1 monoclonal antibody (mAb) treatment (38). In addition, increased FDG uptake in the spleen and bone marrow has also been observed in melanoma mice treated with genetic vaccine adjuvant immunotherapy (39). Thus, visualization of changes in splenic FDG uptake by PET/CT following immunotherapy has the potential to be a suitable predictor.

In our study, spleen metabolic indices were more accurate predictors of ICI prognosis than bone marrow metabolic indices. Seban et al. showed that higher bone marrow glycolytic activities at baseline were associated with poor OS but not treatment responses (12). However, Schwenck et al. showed that baseline bone marrow FDG uptake was significantly higher in responders than in non-responders (38). This may be due to the differences in inclusion criteria among studies, resulting in variations in data on bone marrow metabolism in patients with potential bone metastases (40). Bone marrow glycolytic activity may be affected by bone metastatic lesions, which leads to less confidence in the bone marrow data. However, this was not found in spleen data. The optimal method of bone marrow delineation has not been unified. Considering these, we strictly limited the study of patients to those without bone metastases and excluded all patients with bone metastases in the analysis of bone marrow metabolism. In the current study, delineation of the bone marrow was improved based on previous methods and was easier to standardize (38). In summary, the use of bone marrow data is less controllable and reliable than spleen data. The spleen is a better choice for monitoring the immune system status in secondary lymphoid organs. Furthermore, due to the diversity of various characteristics in the study population, there are currently no standard cut-off values for metabolic parameters. Receiver operating characteristic (ROC) curve analysis grouping results in a large sample gap between the two groups; thus, we have referred to previous methods and used the median as the cutoff value (9, 19).

Overall, using the TMTV and SLR to assess the pretreatment host immune environment and then taking into account the changes in spleen metabolism after treatment is sufficient for assessing the development of an effective immune activation to achieve longer survival. This strategy of using combined biomarkers can better stratify patients and help develop personalized immunotherapy strategies. However, a limitation of this study is that the retrospective study design results in the heterogeneity of the enrolled patients, although it is generally believed that the patient immune status is sufficient to influence the efficacy of immunotherapy. We included patients with NSCLC and SCLC, which is also a deficiency of this study. However, the PET/CT parameters of patients with NSCLC and SCLC are balanced and our results can be applied to lung cancer. Further, considering the complexity of immunotherapy response, there is no final conclusion on which response criteria is preferable to evaluate the treatment response of ICI (32). The immunotherapy responses were assessed using RECIST 1.1 in this study. There is a good agreement between iRECIST and RECIST 1.1 when evaluating immunotherapy. At present, RECIST 1.1 is the main evaluation system in immuno-oncology, and more prospective studies are needed to test response criteria to ICI (41, 42).

Various immune cells mobilized into normal organs interact with tumor cells, which is key to determining treatment outcomes (11). In our results, TMTV representing tumor aggressiveness as a “seed” and SLR representing host immune status as a “soil” could both be visualized and estimated by 18F-FDG PET/CT. Moreover, systemic immune activation guarantees effective immunotherapy responses (10). In addition, the PET community proposed that after immunotherapy, the increased metabolic activity in the morphologically stable lesions may indicate that immune cells are recruited into tumor microenvironment and activated. This increased metabolic activity is resulted by immune activation of lymphoid organs, rather than disease progression (32). Therefore, after ICI treatment, the metabolic status of tumor and lymphoid organ can be evaluated simultaneously to evaluate disease progression and pseudoprogression. We are conducting prospective clinical studies to predict the prognosis of patients treated with anti-PD1 therapy using immune system and systemic immune activation markers monitored by PET/CT. In addition, correlation of the metabolic parameters of the tissue microenvironment with immune cell populations in peripheral blood will be explored. Large-scale PET/CT-based radiomic characterization and texture analysis is also an approach. High-dimensional mineable data through machine learning helps discover the relationship between radiomic signatures and immune signature biomarkers (43). Future studies should also explore the application of the combination of new factors, such as circulating tumor cells and circulating tumor DNA minimal residual disease (44). A recent study has shown that circulating tumor cell count during ICI is associated with the tumor metabolic response in patients with NSCLC, and the combination of circulating tumor cells and MTV can predict the prognosis in these patients (45). Large-scale prospective studies are necessary to explore the predictive value and mechanism of PET/CT metabolic patterns in immunotherapy.

In conclusion, this study established a method for predicting the prognosis of advanced lung cancer with ICI therapy by analysis of the combination of tumor metabolic parameters and secondary lymphoid metabolic parameters on 18F-FDG PET/CT. High TMTV (>168 mL) and spleen metabolism (SLR >1.08) were predictors of poor prognosis, and systemic immune activation indicated by increased splenic FDG uptake post-ICI treatment predicted survival benefits.

## Data availability statement

The raw data supporting the conclusions of this article will be made available by the authors, without undue reservation.

## Ethics statement

The studies involving human participants were reviewed and approved by Institutional review board of Shandong Cancer Hospital and Institute. The patients/participants provided their written informed consent to participate in this study. Written informed consent was obtained from the individual(s) for the publication of any potentially identifiable images or data included in this article.

## Author contributions

PJ, conceptualization, methodology, data curation, visualization, and writing - original draft. MB, data curation and visualization. JL, visualization and investigation. JY, conceptualization and supervision. XM, conceptualization, writing- reviewing and editing, and supervision. All authors contributed to the article and approved the submitted version.

## Funding

This study was supported by National Natural Science Foundation of China (81972864 and 82172720), Science and Technology Support Plan for Youth Innovation Teams of Universities in Shandong Province (2019KJL001), Bethune Translational Medicine Research Foundation for Tumor Radiotherapy (flzh202106).

## Conflict of interest

The authors declare that the research was conducted in the absence of any commercial or financial relationships that could be construed as a potential conflict of interest.

## Publisher’s note

All claims expressed in this article are solely those of the authors and do not necessarily represent those of their affiliated organizations, or those of the publisher, the editors and the reviewers. Any product that may be evaluated in this article, or claim that may be made by its manufacturer, is not guaranteed or endorsed by the publisher.

## Abbreviations

ANZSNM, Australian and New Zealand Society of Nuclear Medicine; BLR, bone marrow-to-liver SUVmax ratio; CT, computed tomography; DCs, dendritic cells; EANM, European Association of Nuclear Medicine; FDG, fludeoxyglucose; ICI, immune checkpoint inhibitor; MDSCs, myeloid-derived suppressor cells; MTV, metabolic tumor volume; NSCLC, non-small cell lung cancer; OS, overall survival; PD1, programmed cell death protein 1; PET, positron emission tomography; PFS, progression-free survival; SLR, spleen-to-liver SUVmax ratio; SNMMI, Society of Nuclear Medicine and Molecular Imaging; SUVmax, maximum standardized uptake value; SUVmean, mean standardized uptake value; TLG, total lesion glycolysis; TMTV, total metabolic tumor volume; Tregs regulatory T cells.
