# Supplementary figures and images for: Tumor metabolic and secondary lymphoid organ metabolic markers on 18F-fludeoxyglucose positron emission tomography predict prognosis of immune checkpoint inhibitors in advanced lung cancer
Source: Front Immunol. 2022 Oct 21;13:1004351. doi: 10.3389/fimmu.2022.1004351 (PMC9634068; doi:10.3389/fimmu.2022.1004351)

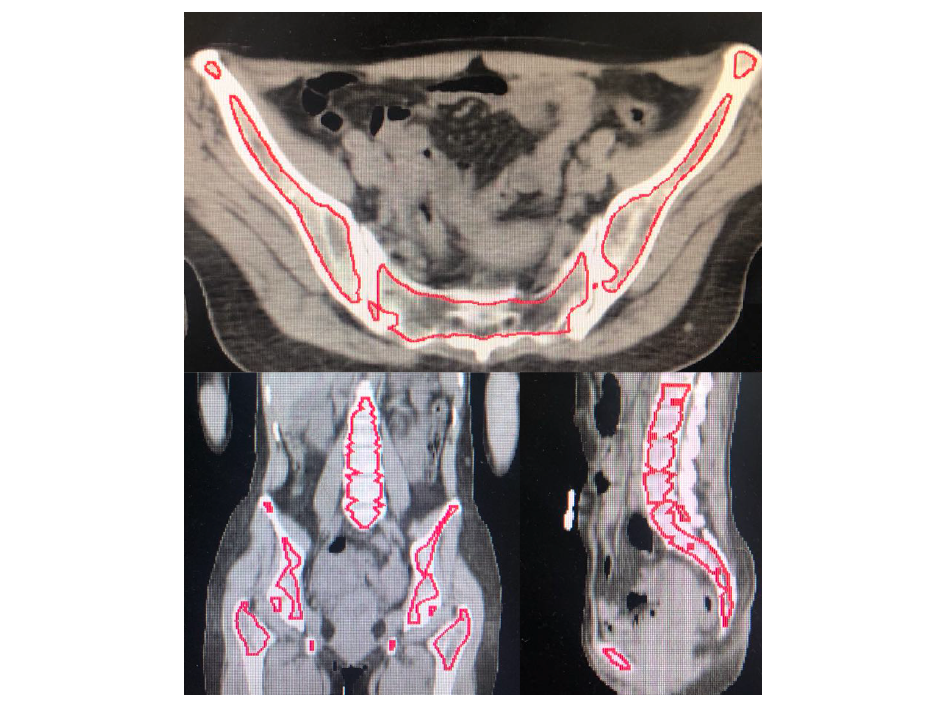

Supplement: Supplementary file 1 [file Image_1.tif]

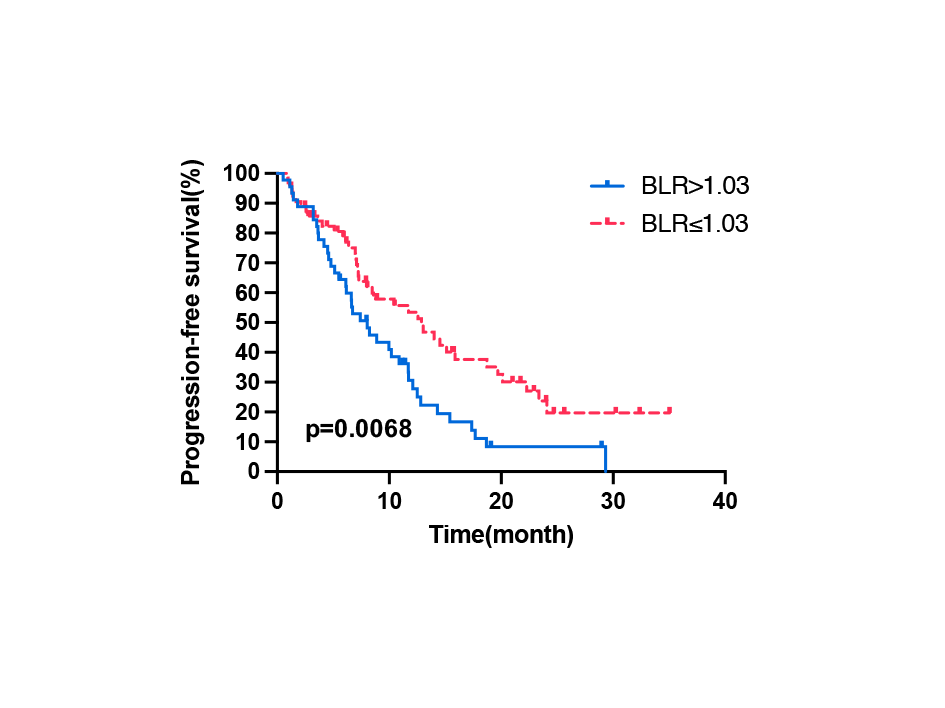

Supplement: Supplementary file 2 [file Image_2.tif]
