# Supplementary material for: Tumor metabolic and secondary lymphoid organ metabolic markers on 18F-fludeoxyglucose positron emission tomography predict prognosis of immune checkpoint inhibitors in advanced lung cancer
Source: Front Immunol. 2022 Oct 21;13:1004351. doi: 10.3389/fimmu.2022.1004351 (PMC9634068; doi:10.3389/fimmu.2022.1004351)
Supplement: Supplementary file 3 [file DataSheet_1.docx]

Supplementary Material

# Supplementary Data

## Supplementary Figures

**Supplementary Figure 1.** Schematic representation of bone marrow delineation

**Supplementary Figure 2.** Kaplan–Meier curves of PFS according to the BLR. PFS: progression-free survival; BLR: bone marrow-to-liver SUVmax ratio

## Supplementary Tables

**Supplementary Table 1.** Comparison of PET/CT metabolic parameters between NSCLC and SCLC

|  | NSCLC | SCLC | P |
| --- | --- | --- | --- |
| Tumor metabolism |  |  |  |
| SUVmax | 11.52±6.13 | 10.39±4.67 | 0.547 |
| SUVmean | 4.58±1.64 | 4.59±1.29 | 0.861 |
| TLG | 448.48±704.51 | 301.00±330.07 | 0.923 |
| TMTV | 177.66±237.69 | 118.71±118.00 | 0.500 |
| Secondary lymphoid organ metabolism |  |  |  |
| SLR | 1.08±0.21 | 1.10±0.19 | 0.454 |
| BLR | 1.04±0.23 | 0.98±0.19 | 0.312 |
| ΔSLR | 0.17±0.68 | -0.05±0.34 | 0.842 |
| ΔBLR | -0.02±0.37 | 0.06±0.31 | 0.619 |

PET/CT: positron emission tomography/computed tomography; NSCLC: non-small cell lung cancer; SUVmax: maximum standardized uptake value; SUVmean: mean standardized uptake value; TLG: total lesion glycolysis; TMTV: total metabolic tumor volume; SLR: spleen-to-liver SUVmax ratio; BLR: bone marrow-to-liver SUVmax ratio.

**Supplementary Table 2.** Univariate analysis of influencing factors of progression-free survival

|  | Progression-free survival |  |
| --- | --- | --- |
|  | HR(95%CI) | P |
| Tumor metabolism |  |  |
| High SUVmax | 1.122(0.741-1.699) | 0.585 |
| High SUVmean | 0.982（0.649-1.487） | 0.932 |
| High TLG | 1.216（0.776-1.907） | 0.393 |
| High TMTV | 1.299(0.841-2.005) | 0.238 |
| Secondary lymphoid organ metabolism |  |  |
| High SLR | 1.387(0.902-2.130) | 0.136 |
| High BLR | 1.847(1.176-2.901) | 0.008 |

HR: hazard ratio; SUVmax: maximum standardized uptake value; SUVmean: mean standardized uptake value; TLG: total lesion glycolysis; TMTV: total metabolic tumor volume; SLR: spleen-to-liver SUVmax ratio; BLR: bone marrow-to-liver SUVmax ratio.

**Supplementary Table 3.** Comparison of change in PET/CT metabolic parameters between response groups

|  | Responders（n=17） | Non-responders(n=24) | P | Long-term survival（n=24） | Short-term survival (n=17) | P |
| --- | --- | --- | --- | --- | --- | --- |
| Tumor metabolism |  |  |  |  |  |  |
| ΔSUVmax | -5.73±7.19 | -0.33±5.72 | 0.01 | -2.92±5.97 | -2.07±8.07 | 0.412 |
| ΔSUVmean | -1.29±1.81 | -0.41±1.96 | 0.068 | -0.97±1.62 | -0.52±2.32 | 0.165 |
| ΔTLG | -142.82±1005.68 | -68.12±514.02 | 0.383 | -101.24±847.77 | -126.83±838.20 | 0.578 |
| ΔTMTV | -4.60±230.11 | -59.00±249.48 | 0.979 | 10.85±204.78 | -103.21±275.48 | 0.161 |
| Secondary lymphoid organ metabolism |  |  |  |  |  |  |
| ΔSLR | 0.45±0.89 | -0.75±0.23 | 0.02 | 0.31±0.63 | -0.09±0.62 | 0.005 |
| ΔBLR | 0.10±0.42 | -0.08±0.29 | 0.117 | 0.07±0.40 | -0.14±0.22 | 0.053 |

SUVmax: maximum standardized uptake value; SUVmean: mean standardized uptake value; TLG: total lesion glycolysis; TMTV: total metabolic tumor volume; SLR: spleen-to-liver SUVmax ratio; BLR: bone marrow-to-liver SUVmax ratio.
